# Community Health Impacts of Pesticide Exposure: Pathways, Vulnerable Populations, and Public Health Responses

**DOI:** 10.3390/ijerph23070889

**Published:** 2026-07-10

**Authors:** Turki Kh. Faraj

**Affiliations:** 1 Department of Soil Science, College of Food and Agricultural Sciences, King Saud University, Riyadh 11451, Saudi Arabia; talasiri@ksu.edu.sa; Tel.: +966-505757249; 2 Prince Sultan Institute for Environmental, Water and Desert Research, King Saud University, Riyadh 12372, Saudi Arabia

**Keywords:** agricultural workers, biomonitoring, environmental health, food safety, groundwater contamination, integrated pest management, public health policy, vulnerable populations

## Abstract

**Highlights:**

**Public health relevance—How does this work relate to a public health issue?**

- Pesticide exposure is a community-level public health issue because exposure occurs among agricultural workers and among families, children, pregnant women and fetuses, elderly individuals, rural residents, and non-farm populations living near treated areas.
- This review highlights multiple exposure pathways, including occupational, take-home, residential, dietary, drinking-water, airborne, and public-space exposure, showing that pesticide-related health risks extend beyond direct pesticide applicators.

**Public health significance—Why is this work of significance to public health?**

- Pesticide exposure is associated with acute poisoning and a wide range of chronic health outcomes, including neurological, respiratory, reproductive, developmental, endocrine, metabolic, cardiovascular, dermatological, immunological, teratogenic, mutagenic, and carcinogenic effects.
- The problem is often more pronounced in low- and middle-income countries and underserved rural communities due to poverty, weak regulatory frameworks, unsafe storage and disposal practices, limited access to protective equipment, inadequate surveillance, and reduced access to healthcare.

**Public health implications—What are the key implications or messages for practitioners, policy makers and/or researchers in public health?**

- Public health responses should prioritize community education, safer pesticide handling and storage, worker protection, drift reduction, residue monitoring in food and water, biomonitoring, and improved surveillance of pesticide-related illness.
- Policy makers and researchers should strengthen regulation of hazardous pesticides, promote integrated pest management, improve exposure assessment methods, and develop community-level indicators to better identify and protect vulnerable populations.

**Abstract:**

Pesticide use remains important in modern agriculture, vector control, and household pest management. However, exposure to pesticide active ingredients and residues remains a persistent public health concern. In addition to active ingredients, people may also be exposed to other constituents of commercial pesticide formulations, such as adjuvants and solvents, which can influence overall toxicity and health outcomes. These exposures may occur among direct applicators and may also affect other populations through contaminated air, water, soil, food, clothing, and household surfaces. This narrative review examines pesticide exposure from a community health perspective, emphasizing occupational, para-occupational, residential, dietary, drinking-water, airborne, and cumulative exposure pathways. It highlights vulnerable groups, including agricultural workers, children, pregnant women, women in agricultural communities, older adults, people with chronic illness, and marginalized rural populations. Evidence reviewed in this article links pesticide exposure with acute poisoning, respiratory effects, neurobehavioral and neurodevelopmental outcomes, cancer-related risks, reproductive and developmental effects, endocrine and metabolic disruption, cardiovascular outcomes, dermatological reactions, immune dysregulation, and biomarker-based subclinical changes. Beyond disease endpoints, pesticide exposure may also affect household income, education, mental well-being, food security, livelihoods, and intergenerational health. Major challenges include weak exposure assessment, underreporting, limited biomonitoring in low- and middle-income settings, and inconsistent community-level indicators. Strengthening surveillance, risk communication, integrated pest management, safer storage and disposal, protective regulation, and community-centered biomonitoring is essential to reduce pesticide-related health burdens.

## 1. Introduction

Pesticides are natural or chemically synthesized compounds used to control weeds, insects, fungi, rodents, and other pest-related problems [1,2]. Global pesticide consumption in 2023 reached 3.73 million metric tons of active ingredients, representing a 14% increase over the past decade [3]. The World Health Organization (WHO) reports that over 1000 pesticides are in use globally [4]. They play a key role in agriculture by reducing crop losses and improving both yield and quality of food [5]. Pesticides contribute to food production by improving the quality and quantity of agricultural products. They can reduce insect damage and contamination and may help stabilize food prices by improving crop yields. In addition to agricultural applications, pesticides are also used to control disease vectors that transmit human diseases such as dengue, malaria, West Nile virus disease, and Lyme disease [6].

However, despite these benefits, pesticide use is associated with significant risks to human health and the environment. Approximately 3 billion kilograms of pesticides are used each year globally and only about 1% effectively reach target pests [7]. The remaining amount is released into the environment, contributing to environmental contamination, and potential adverse health effects [5]. As a result, many review articles have been written regarding the negative effects of pesticides on human health. Although a large number of studies reported pesticide-related risks, most studies have concentrated on occupational exposure, environmental contamination, or specific health impacts, including respiratory issues, carcinogenicity, or neurotoxicity [1,5,8,9]. Several studies have examined the adverse effects of pesticide exposure among vulnerable groups, particularly women, children, and developing fetuses exposed during pregnancy [10,11,12]. Prenatal exposure has been associated with adverse birth and developmental outcomes, including low birth weight, preterm birth, congenital abnormalities, and later neurodevelopmental effects [13,14]. Therefore, fetal exposure represents an important public-health concern that is distinct from both maternal exposure and postnatal childhood exposure. Yet relatively few studies have analyzed and reported these impacts from a community health perspective. Community can be described as “a group of people with diverse characteristics who are linked by social ties, share common perspectives, and engage in joint action in geographical locations or settings” [15]. In the context of this review, a community health perspective refers to populations sharing common environmental exposures, such as households, neighbors, schools, and non-farm residents, as well as vulnerable rural populations and occupational exposure.

Pesticide exposure can occur through multiple pathways i.e., direct exposure in occupational, household and agricultural settings, and indirect exposure through contaminated environmental media [5,16]. People who work directly and frequently with pesticides during manufacturing, transportation, preparation, and specific users in the workplace, such as exterminators of house pests, are considered to have occupational exposure [1,17]. Occupational exposure in the agricultural sector is common among farmers and farmworkers [1,18]. Human and environmental exposure to pesticides can also occur through non-occupational pathways. Residential exposure can occur when farm workers bring pesticide-contaminated articles into their home, live in proximity to a pesticide-applied farm, apply pesticides in or around the home, and use pesticides at home, garden, humans (scabies and lice), or pets (ticks and fleas). However, delayed household exposure can occur through contact with residual pesticides on various surfaces, including beds, clothing, dust, food, or spraying equipment, or through inhalation of residual air concentrations [18,19]. Dietary exposure mainly occurs through dietary intake while eating foods and drinking water that are contaminated with pesticides [19,20]. These facts confirm that pesticide and pesticide residue exposure occur in several ways, not only farmers or farm workers, but also almost all human beings can be exposed, particularly women and children [11,12]. Therefore, a comprehensive understanding of pesticide exposure and health impacts at the community level is essential. Sustainable pesticide management should also be integrated within broader environmental health and food security strategies, particularly in vulnerable agricultural and water-scarce regions.

This review highlights evidence from the published literature on the impacts of pesticide use on community health. It analyzes how exposure occurs through multiple pathways, including occupational, para-occupational, residential, and dietary pathways. This review also focuses on vulnerable groups such as agricultural workers, their families, women, children, non-farm rural residents, and other community groups who may be indirectly exposed. In addition, it explores community-level health impacts related to pesticide exposure, along with social and health-system determinants that influence pesticide exposure and risk. The review also analyzes relevant prevention strategies and policy responses to reduce exposure and prevent adverse health effects at the community level.

## 2. Review Approach

This review brings together peer-reviewed literature on pesticide exposure pathways, vulnerable populations, community-level health effects, exposure assessment, biomonitoring, and public health responses. The relevant literature was identified through searches of major scientific databases, including Web of Science, Scopus, PubMed, Google Scholar, and ScienceDirect. The searches were conducted using combinations of keywords related to pesticide exposure and community health, including “pesticide exposure”, “community health”, “occupational exposure”, “residential exposure”, “take-home exposure”, “dietary exposure”, “drinking water contamination”, “spray drift”, “vulnerable populations”, “children”, “pregnant women”, “farm workers”, “biomonitoring”, “acute pesticide poisoning”, “chronic health effects”, “public health intervention”, and “pesticide policy”.

Studies were considered relevant if they addressed pesticide exposure in human populations, reported exposure pathways, examined health outcomes associated with pesticide exposure, discussed vulnerable or exposed community groups, or evaluated prevention, monitoring, or policy responses. Priority was given to review articles, systematic reviews, epidemiological studies, biomonitoring studies, public health reports, and policy-relevant documents that provided evidence on community-level exposure and health impacts. Studies focusing only on toxicological mechanisms without relevance to human or community exposure were used selectively when they supported interpretation of health effects. Articles not written in English, studies without clear relevance to human health, and publications lacking sufficient methodological or contextual information were excluded.

The literature was organized thematically rather than statistically because the included studies varied widely in design, population, exposure assessment methods, pesticide classes, and reported health outcomes. Evidence was synthesized under major themes, including exposure pathways, vulnerable populations, acute and chronic health effects, broader community consequences, challenges in exposure assessment, biomonitoring gaps, and public health and policy responses. Where possible, findings from epidemiological and biomonitoring studies were used to support evidence from broader reviews.

## 3. Framing Pesticide Exposure as a Community Health Problem

### 3.1. What Is Meant by “Community Health” in Pesticide Research?

Before discussing community health, it is essential to identify the concept of “community”. A community is commonly described as a group of people living in a defined geographical area who share similar values, interests, and social relationships. They may also have a sense of identity or belonging and organize themselves to meet their common needs [15,21]. There are various population groups based on gender and age. The community health term is used to describe health services provided in the community, such as those delivered in community health centers or by health visitors, rather than in institutional settings like hospitals [22]. It refers to the overall health status of a defined group of people and the actions taken to promote, protect, and maintain their health [23]. The community well-being term can be defined as “the combination of social, economic, environmental, cultural, and political conditions identified by individuals and their communities as essential for them to flourish and fulfil their potential” [24]. There are many physical, social, and cultural factors that affect community health [23].

In pesticide research, community health is affected through overlapping environmental, occupational, social, and health-system pathways. A community perspective therefore moves beyond individual exposure and considers shared risks, such as contaminated household dust, drift from nearby farms, pesticide residues in food and water, unsafe storage practices, limited access to health care, and weak regulatory protection. This framing is important because the same exposure source can affect workers, family members, neighbors, schools, consumers, and vulnerable groups in different but connected ways.

### 3.2. Types of Communities Affected

In the agricultural sector, farmers, farm workers, greenhouse workers, florists, and professional pesticide applicators are generally at higher risk of pesticide exposure than many other occupational groups [17,19]. Exposure can also occur among workers involved in pesticide production, transport, mixing, and application, as well as among public health workers who use pesticides for vector or household pest control [17]. Non-farm residents living near agricultural areas, together with their family members, may also experience higher pesticide exposure than the general population because of spray drift, volatilization, and contaminated household dust [25]. Rural areas have a greater concern due to the proximity to pesticide application sites [26]. Plantation communities, including workers and residents living in large-scale tea, fruit, horticulture, or other commercial crop estates, may experience repeated pesticide exposure through occupational and environmental exposure pathways [27,28,29]. A transitional zone with mixed characteristics of both urban and rural environments is considered a peri-urban area. Peri-urban communities are exposed to pesticides due to pesticide applications in neighboring gardens, household settings, and dietary exposure [30]. At the residential level, due to the application of household pest treatments and regular household cleaning, urban, and non-farmworker communities are also exposed to pesticides [19,31]. Communities residing near pesticide storage facilities and waste disposal sites are also vulnerable to chronic exposure to pesticides [32]. Human populations, livestock, and wildlife that use groundwater for drinking can be exposed to pesticides through contaminated water [33]. Pesticide contamination can affect both groundwater and surface-water resources that support rural, urban, agricultural, and industrial needs [34]. Surface drinking-water supplies may also be contaminated through spray drift, agricultural runoff, industrial discharges from pesticide production facilities, and improper pesticide storage or disposal. The general community can be exposed to pesticides through the consumption of contaminated foods containing pesticide residues, including fruits and vegetables, dairy milk, coffee, cereal grains, and fish [19,35,36]. These findings suggest that pesticide exposure is widespread. It affects diverse populations across rural, peri-urban, and urban environments through various occupational and non-occupational exposure pathways.

### 3.3. Why Community Exposure Is Often Overlooked

Community exposure is often overlooked because pesticide research has traditionally focused on applicators, farmworkers, or single health outcomes. Non-occupational exposure among family members, nearby residents, children, and consumers is more difficult to detect because it may occur at lower doses, through mixed pathways and over long periods. These exposures are also less likely to be recorded in occupational health systems or poisoning surveillance databases.

Additional barriers include limited biomonitoring capacity, poor documentation of pesticide use at the household level, incomplete reporting of acute poisoning, and weak recognition of gendered and social dimensions of exposure. As a result, the public health burden of pesticide use may be underestimated, particularly in rural and low-resource communities.

## 4. Pathways of Pesticide Exposure at the Community Level

**Figure 1:**
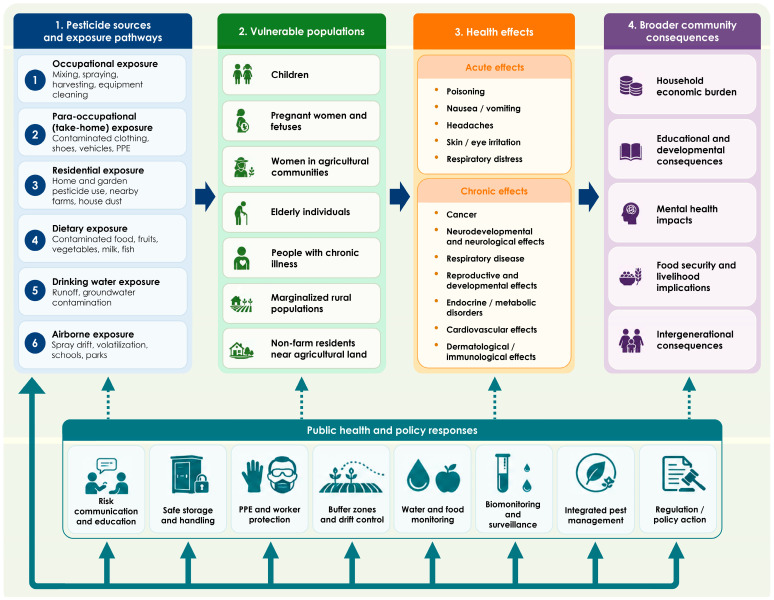
Conceptual overview of pesticide exposure pathways, vulnerable populations, health effects, and public health responses.

Pesticides are widely used worldwide, resulting in exposure among millions of people through occupational and environmental pathways [37]. They are used not only in agriculture but also in commerce, public health, and household settings [16]. As outlined in Figure 1, pesticide exposure occurs through occupational, para-occupational (take-home), residential, dietary, drinking-water, and airborne/public-space pathways, disproportionately affecting vulnerable populations and contributing to acute and chronic health effects, broader household and community consequences, and intergenerational impacts. Public health and policy interventions are intended to reduce exposure and its associated health burden.

### 4.1. Direct Occupational Exposure Within the Community

Occupational exposure to pesticides primarily affects individuals employed in pesticide manufacturing, agriculture, forestry, and pest-control services, with the highest exposure occurring among workers who directly and routinely handle these compounds, including formulators, mixers, loaders, sprayers, and farm workers [1,37,38,39,40]. Exposure may occur during pesticide production, transportation, preparation, mixing, loading, application, harvesting in recently treated fields, and equipment cleaning [1,38,39]. Exposure may be further increased by unsafe application practices, poorly maintained equipment, inadequate use of personal protective equipment, unsafe storage, transfer of pesticides into household containers, and reuse of pesticide containers for food or water storage [19,37]. Sprayers, including those using knapsack sprayers, may face especially high exposure because of their close contact with pesticide mixtures and application equipment [41]. Greenhouse workers and florists may also be exposed during pesticide use on flowers and foliage [19].

Occupational pesticide exposure depends on several factors, including the frequency, intensity, method of application, duration, physical form, environmental conditions, and safety practices such as the use of personal protective equipment, as well as the physicochemical and toxicological properties of the pesticides used [1]. The physical state of pesticide formulations influences the extent of exposure. For instance, liquid formulations are more prone to splashing, leading to direct skin contact. In contrast, solid formulations may generate dust during handling, which results in exposure to the face and eyes. Environmental conditions such as temperature and humidity during application can affect pesticide volatility, which can increase absorption level [19].

### 4.2. Para-Occupational or Take-Home Exposure

Para-occupational or take-home pesticide exposure is an indirect form of exposure that occurs when workers unintentionally transfer pesticide residues from the workplace to their homes on their skin, hair, clothing, shoes, or vehicles [42]. Para-occupational pathways are complex, as they involve both direct contact such as inhalation of pesticide drift and indirect exposure through residues in carpets, vehicles, and laundry. The main para-occupational exposure pathways include the transfer of pesticide residues from worker boots, clothing, vehicles, and equipment into the home environment [43]. These residues may contaminate floors, carpets, laundry areas, and household dust, increasing the risk of exposure among family members [43]. Family members of pesticide applicators experience considerable exposure, which can occur due to accidental pesticide spills, leakages from equipment, improper equipment handling, and failures to comply with safety guidelines [1]. This exposure pathway was initially identified in 1995 in a study by the National Institute for Occupational Safety and Health (NIOSH), which reported contamination of homes with workplace chemicals, including pesticides, across several countries. Later research in high-income countries further showed that pesticides, such as herbicides, can accumulate in homes, increasing the risk of exposure among family members [42]. Although personal protective equipment (PPE) is important for reducing pesticide exposure in the workplace, contaminated PPE may also contribute to take-home exposure if it is brought into the home. Work boots, gloves, and clothing can carry pesticide residues from treated fields or work areas into household environments, where family members may come into contact with contaminated surfaces or dust [42].

### 4.3. Residential Exposure

Another source of pesticide exposure is the residential environment. It occurs due to the usage of pesticides at home, in the garden, or the lawn [19]. One method of residential pesticide exposure occurs due to the proximity of non-farmworker residents to agricultural lands, where frequent and intensive pesticide application occurs [25]. Residential exposure may occur through several household and outdoor pesticide uses, including treatment for fleas, ticks, ants, flies, cockroaches, bees, wasps, and hornets. It may also result from professional pest-control treatments, garden or lawn pesticide applications, and pesticide residues that accumulate in carpet dust. Treatments for ants, flies, or roaches are also associated with residential pesticide exposure [19]. Spray drift and volatilization of pesticides beyond the pesticide-treated region are major residential pesticide exposure pathways [25]. Previous studies reported that, compared to non-farm homes, the pesticide concentrations in residential dust of farm homes are higher for some reasons, including proximity to treated fields [44]. Regular household cleaning, such as vacuuming at least once a week, is related to the lower atrazine pesticide concentrations in residential dust [19]. Although occupational and take-home pathways are primary pesticide exposure contributors, evidence increasingly shows that farmworkers and their families residing near agricultural lands are highly exposed to a wider range of pesticides compared to the general population [25]. Previous studies showed that pet dogs can also act as a pathway for pesticide exposure, with homes containing dogs showing higher levels of pesticides such as atrazine, dacthal, and chlorpyrifos [19].

### 4.4. Dietary Exposure

When direct pesticide application to food crops and harvesting before the recommended waiting period has elapsed, it can result in pesticide residues in human-consumed food products [45,46]. The general population is exposed to pesticides mainly through eating contaminated food and drinking contaminated water [19,35]. Pesticide application during food production and storage frequently results in pesticide residue contamination of food products, especially fruits and vegetables, which can remain even after washing [47]. Fruits and vegetables are the main dietary contributors to exposure to pesticide residues while also providing substantial nutritional benefits and contributing to the prevention of major chronic diseases [36]. Evidence from Saudi Arabia also highlights the importance of dietary pesticide exposure as a community health concern [48]. A study conducted in the Riyadh region (Al-Kharj Province) detected pesticide residues in commonly consumed leafy vegetables, including lettuce, parsley, coriander, spinach, dill, mint, and green onions. The study identified residues from several pesticide groups using gas chromatography–mass spectrometry (GC-MS), emphasizing that food consumption can represent an important non-occupational pathway of pesticide exposure within the community. Various pesticide residues have been detected in several food products, including malathion in vegetables and leafy vegetables, thiamethoxam in coffee leaf extract, DDT in wheat, dairy milk, and cereal grains, organochlorine pesticides in cow milk and human milk, and different HCH types in fish, dairy milk, maize, cowpea, cheese, and wheat [19]. Pesticide residues can also be transmitted to breast milk and baby food [46]. These findings support the need for continuous food safety monitoring, residue surveillance, and strengthened public health measures to reduce dietary exposure risks in agricultural and urban populations.

### 4.5. Drinking Water Exposure

Pesticides can enter aquatic systems through surface and subsurface hydrological pathways, primarily via surface runoff and leaching to groundwater. Their movement into surface water is primarily through agricultural runoff, drift, agricultural stormwater discharges, return flow from irrigated fields, erosion, groundwater exchange, drainage inflow, and industrial wastewater drainage [49,50,51]. Pesticides can be found in surface water, groundwater, and even drinking water [52]. Inadequate handling practices, including improper sprayer filling, equipment washing, disposing of packaging materials, and cleaning spraying equipment, can further contribute to this contamination [49]. Agricultural drainage systems promote the transport of pesticides by allowing rapid movement from soil and shallow groundwater into surface water bodies. Polar pesticides are generally more soluble. Therefore, they are more quickly transported to surface and groundwater, while non-polar pesticides tend to accumulate in sediments and suspended particles. Only highly persistent and mobile pesticides can transfer into the groundwater, as many compounds are retained within upper soil layers [52]. Low concentrations of pesticides in water can bioaccumulate through the food chain by entering aquatic organisms [50]. Previous studies have reported that the presence of pesticides in drinking water originating from surface water sources and the pesticide concentration is increasing during the season of intensive pesticide application [52]. Application of ultra-low-volume insecticide aerosols for mosquito control can contaminate water resources. Dust can serve as an additional pathway for drinking water contamination, while pesticides with high volatility can be transported through the air and contribute to the contamination of water systems [53]. Saudi Arabia provides additional evidence regarding environmental and community exposure to pesticides. A study conducted in the Al-Kharj area investigated groundwater contamination associated with intensive pesticide use in arid agricultural regions and detected multiple pesticide residues in groundwater samples. Several detected compounds exceeded recommended safety limits, highlighting the importance of groundwater monitoring, environmental risk assessment, and sustainable pesticide management in water-scarce communities [54].

### 4.6. Community Exposure Through Air and Public Spaces

Airborne pesticide contamination can occur mainly through spray drift and volatilization. Spray drift carries pesticide droplets away from the target application area, while volatilization allows pesticides to evaporate from soil or plant surfaces and move through the air, potentially exposing nearby communities. Spray drift refers to the movement of pesticide droplets beyond the target area during or soon after application. Furthermore, factors such as operating pressure, nozzle type, weather conditions, and spray height influence spray drift [55]. Up to 88.8% of applied pesticides may be lost as drift in the form of droplets, particles, or vapor. These pesticidal droplets have the ability of extreme persistence, which can find every corner, such as targeting and on-target regions. And it is estimated that approximately 97% of applied pesticides have a negative effect on non-targeted organisms, due to their wide spreading capacity and higher persistence capacity [46]. Volatilization from soil and plant surfaces can represent a major emission pathway, sometimes responsible for a large proportion of the applied pesticide over extended periods. Emissions from plant surfaces may exceed those from soil, highlighting its importance in long-term emissions. While spray drift typically results in local deposition, volatilized pesticides can transport over longer distances in the atmosphere [55].

### 4.7. Mixed and Cumulative Exposure Pathways

At the community level, pesticide exposure rarely occurs through a single route [5,19,25,42,44,50]. Workers and residents may simultaneously experience dermal, inhalation, dietary, drinking-water, household-dust, and take-home exposures. These pathways may also involve multiple pesticide classes, mixtures of active ingredients, adjuvants, and degradation products. Cumulative exposure is therefore shaped by the frequency of application, proximity to treated areas, household hygiene, food consumption patterns, water sources, age, occupation, and use of protective measures.

This mixed-exposure reality is particularly important for children, pregnant women, older adults, and people with chronic diseases because low-level exposures from several sources may combine with physiological susceptibility and social vulnerability [12,56,57,58,59,60,61]. Future community studies should therefore assess cumulative exposure patterns rather than treating occupational, residential, environmental, and dietary pathways as isolated categories. Community-level pesticide exposure occurs through multiple overlapping pathways. These pathways, affected groups, and possible prevention points are summarized in Table 1.

## 5. Determinants of Community Vulnerability

Community vulnerability to pesticide exposure is shaped by a combination of social, economic, regulatory, health-system, behavioral, cultural, geographic, and environmental factors. These determinants influence who is exposed, the intensity and duration of exposure, the ability to adopt protective measures, and the likelihood of receiving timely diagnosis, treatment, and prevention support. Therefore, pesticide-related community health risk should not be understood only as a toxicological issue, but also as a broader public health problem linked to livelihood, governance, infrastructure, education, and environmental conditions [5,10,16].

### 5.1. Socioeconomic Determinants

Socioeconomic conditions strongly influence pesticide exposure and vulnerability at the community level. Poverty is one of the major determinants because low-income farmers and agricultural workers often have limited capacity to purchase safer pesticide products, appropriate personal protective equipment, or alternative pest management technologies [5,62]. In many rural settings, pesticide use is closely linked to agricultural productivity and household income, making it difficult for farmers to reduce pesticide use even when they are aware of possible health risks [63]. This creates a cycle in which communities remain dependent on pesticides for crop protection and livelihood security while also carrying the health and economic burden of exposure.

Dependence on agriculture for livelihood increases vulnerability because farming households may experience repeated exposure across multiple settings, including fields, homes, storage areas, and transport routes. Farmers and farm workers may be exposed during mixing, loading, spraying, harvesting in recently treated fields, and cleaning contaminated equipment [1,17,19]. Their family members may also be exposed indirectly through take-home residues on clothing, shoes, vehicles, and personal protective equipment [42,43]. Thus, agricultural dependence can convert occupational exposure into a household and community-level exposure problem.

Low educational level and limited access to training further increase vulnerability. Several studies have shown that inadequate knowledge of pesticide toxicity, label instructions, correct dosage, safe handling, and proper disposal contributes to unsafe pesticide practices [5,64]. Farmers with limited literacy may have difficulty understanding pesticide labels, hazard symbols, application instructions, and protective measures, especially when products are repackaged or sold without clear instructions [65]. Low educational levels may also reduce awareness of chronic health risks as many pesticide-related outcomes, such as cancer, respiratory disease, neurological effects, reproductive disorders, and endocrine disruption, may not appear until long after exposure [5,16,31].

Financial inability to adopt safer alternatives is another important determinant. Although integrated pest management, biological control, safer pesticide formulations, improved equipment, and protective clothing may reduce exposure, these options can be expensive or difficult to access for smallholder farmers and low-income communities [62,66]. In addition, farmers may continue to use hazardous products when they are cheaper, more available, or perceived as more effective against pests [5]. Therefore, socioeconomic disadvantage can increase both pesticide dependence and exposure intensity.

### 5.2. Regulatory and Governance Determinants

Regulatory and governance conditions strongly affect community exposure to pesticides. Weak pesticide regulation can allow highly hazardous pesticides to remain available in agricultural markets, especially in settings where registration, import control, sales monitoring, and product labelling are insufficient [5,67]. In such contexts, communities may be exposed to pesticides that are banned or restricted elsewhere due to their toxicity, persistence, or environmental mobility.

Poor enforcement of existing regulations can also increase risk. Even when pesticide laws exist, inadequate inspection, limited market surveillance, informal sales, and poor control over pesticide application may allow unsafe products and unsafe practices to continue [5,68]. Weak enforcement may contribute to excessive application, use of inappropriate products, spraying close to homes or schools, failure to observe re-entry intervals, and unsafe disposal of containers [5,64]. These practices increase exposure not only among applicators but also among nearby residents, children, and other community members.

Illegal, counterfeit, unlabeled, or unsafe pesticide products are particularly problematic because users may not know the active ingredient, toxicity class, correct dose, or required protective measures. Kromhout [65] highlighted that exposure assessment and risk management are especially difficult in settings where unauthorized pesticides, repackaging, poor labelling, and language or literacy barriers are common. These problems reduce the effectiveness of risk communication and increase the possibility of misuse, accidental poisoning, and environmental contamination.

Limited residue monitoring in food, water, soil, and human biological samples also increases community vulnerability. Pesticide residues may occur in fruits, vegetables, grains, dairy products, fish, drinking water, groundwater, and surface water [19,35,36,50,52]. Without routine residue monitoring, contaminated food or water may remain undetected and communities may continue to experience chronic low-level exposure. Limited monitoring also prevents regulators from identifying high-risk regions, high-risk products, or unsafe farming practices [67,68].

Weak disposal and storage policies further contribute to exposure. Empty pesticide containers may be discarded in fields, reused for household purposes, or stored near homes, food, water, and children [64,69]. Unsafe storage increases the risk of accidental poisoning, especially among children, while improper disposal may contaminate soil and water resources [12,49]. Therefore, pesticide governance should include not only product registration but also clear policies for storage, transport, container collection, waste disposal, residue monitoring, and community education. In addition, legal and regulatory frameworks should consider the protection of non-farm residents who may be affected by pesticide drift from nearby agricultural activities. In some jurisdictions, legal protections for farming activities, such as “Right to Farm” laws, may limit the ability of affected residents to seek remedies for pesticide drift or exposure unless negligence, label violation, or intentional misconduct can be demonstrated. This highlights the need for clearer legal accountability, stronger drift-prevention rules, and better mechanisms to protect neighboring communities from involuntary pesticide exposure.

### 5.3. Health-System Determinants

Health-system capacity plays a central role in determining the severity and visibility of pesticide-related community health impacts. Limited poison centers and emergency response systems can delay diagnosis and treatment of acute poisoning. Acute pesticide poisoning may develop rapidly after exposure and can involve gastrointestinal, respiratory, neurological, dermatological, and cardiovascular symptoms [2,5]. In communities where emergency care is distant or unavailable, poisoning cases may progress to severe outcomes before medical support is received.

Poor diagnostic capacity also contributes to under recognition of pesticide-related illness. Symptoms such as headache, dizziness, nausea, skin irritation, respiratory problems, and fatigue may be nonspecific and may not be correctly attributed to pesticide exposure [5,70]. Chronic outcomes, including cancer, neurological disease, respiratory disease, endocrine disruption, reproductive disorders, and cardiovascular disease, are even more difficult to link to pesticide exposure because they may develop over long periods and may involve multiple risk factors [16,31,71]. Limited access to laboratory testing, occupational history assessment, and trained healthcare workers can therefore lead to misclassification or missed diagnosis.

Weak surveillance systems are another major determinant of vulnerability. Underreporting of pesticide poisoning is widely recognized, especially in developing countries where reporting infrastructure, occupational health systems, and quality control in data collection may be limited [10,68]. Many mild, chronic, household, and non-occupational cases are not captured by surveillance systems, leading to underestimation of the true disease burden [10,31]. This underreporting reduces the ability of public health authorities to identify exposed communities, evaluate prevention strategies, and regulate high-risk products.

Lack of biomonitoring further limits community protection. Biomonitoring can provide direct evidence of internal pesticide exposure through biological samples, such as urine, blood, or other tissues and can help identify subclinical effects before disease becomes clinically visible [8,56]. However, biomonitoring is often limited by cost, technical complexity, short biological half-lives of some pesticides, low-dose detection challenges, and the need for specialized laboratories [8,67]. In many low-resource settings, the absence of routine biomonitoring means that exposure remains invisible until acute poisoning or chronic disease is reported.

### 5.4. Behavioral and Cultural Determinants

Behavioral and cultural practices strongly influence pesticide exposure within communities. Risk perception is a key determinant because people may continue unsafe practices if they underestimate pesticide toxicity, believe that exposure is unavoidable, or prioritize crop protection over health risks [72,73]. In some farming communities, pesticide use may be normalized as an essential part of agriculture, especially where pest pressure is high and livelihoods depend on crop yield [63]. Low risk perception may also reduce the use of protective equipment, safe storage, and correct disposal practices.

Unsafe storage is a common behavioral determinant of exposure. Pesticides stored inside homes, kitchens, bedrooms, or areas accessible to children increase the risk of accidental poisoning and chronic household exposure [12,19]. Storage near food, water, animal feed, or household items can also increase the chance of contamination. In rural communities, inadequate storage may be linked to limited housing space, lack of storage facilities, poor awareness, or weak regulation [64,69].

Reuse of pesticide containers is a particularly dangerous practice. Containers may retain pesticide residues even after washing and their reuse for water, food, cooking materials, or other household items can create direct ingestion and dermal exposure risks [19,69]. In addition, former pesticide storage or handling areas may also remain contaminated and should not be used for the storage of food or feed crops due to the potential for residual contamination and chronic exposure risks. This practice is often associated with poverty, limited access to safe containers, and lack of awareness of residual toxicity. It also reflects the need for stronger container collection and disposal systems.

Lack of protective equipment is another important contributor to exposure. Farmers and applicators may not use gloves, masks, boots, goggles, or protective clothing due to cost, discomfort in hot climates, limited availability, or lack of training [5,17,64]. Even when personal protective equipment is used, it may become a source of take-home exposure if contaminated clothing, boots, or equipment are brought into the home or washed with family clothing [42,43].

Household practices can increase secondary exposure among family members. Washing contaminated work clothes with household laundry, storing pesticide containers indoors, allowing children to play near treated fields, keeping contaminated shoes inside homes and transporting pesticides in family vehicles can transfer residues from occupational settings to domestic environments [42,43]. Regular cleaning may reduce some residential dust residues, but households near treated fields or with occupational pesticide use may still have elevated pesticide levels in indoor dust [19,44].

### 5.5. Geographic and Environmental Determinants

Geographic and environmental conditions strongly shape pesticide exposure at the community level. Proximity to agricultural fields is one of the most important determinants for non-farm residents. People living near pesticide-treated areas may be exposed through spray drift, volatilization, contaminated dust, and residues transported into homes [25,44]. Schools, homes, and public spaces located close to agricultural land may therefore experience exposure even when community members are not directly involved in pesticide application [25,74].

Dry and windy conditions can increase pesticide drift and airborne transport. Spray drift occurs when pesticide droplets move beyond the target area during or soon after application, and it is influenced by wind speed, spray height, nozzle type, operating pressure, and weather conditions [55]. Volatilization from soil and plant surfaces may also contribute to longer-distance atmospheric transport, especially for pesticides with higher volatility [55]. In dry environments, dust may serve as an additional pathway for pesticide movement and may contribute to contamination of indoor spaces, water sources, and public areas [53].

Use of contaminated irrigation or drinking water can increase exposure in both agricultural and domestic settings. Pesticides may enter water systems through runoff, leaching, erosion, drainage flows, stormwater discharge, equipment washing, and improper disposal of containers [49,50,52]. Communities relying on groundwater, wells, or surface water sources may be vulnerable when monitoring and treatment systems are limited. Contaminated water can affect not only human populations but also livestock, aquatic organisms, and food systems [33,34].

Remote rural settings with poor access to healthcare can intensify the health burden of pesticide exposure. In such areas, emergency treatment for acute poisoning may be delayed, chronic symptoms may go undiagnosed, and preventive education may be limited [5,10,68]. Remote communities may also have weaker access to protective equipment, safe disposal services, clean water, and regulatory inspection. As a result, geographic isolation can increase both exposure and the severity of health consequences. Additional evidence from arid agricultural systems further supports the role of environmental conditions in shaping pesticide exposure. For example, a study from the Al-Kharj region of Saudi Arabia reported pesticide residues in vegetable-growing soils, indicating contamination associated with intensive agrochemical use. These findings are particularly relevant in arid regions, where low rainfall, high evaporation, limited soil organic matter, and pressure on groundwater resources may influence pesticide persistence, mobility, and long-term environmental risk. Such contamination may affect soil quality, groundwater sustainability, and human health through non-dietary environmental exposure pathways, emphasizing the need for sustainable pesticide management and long-term environmental monitoring in arid agricultural landscapes [75].

### 5.6. Inequities in Low- and Middle-Income Countries

The burden of pesticide exposure is often greater in low- and middle-income countries because hazardous pesticide use, weak oversight, limited health protections, and poor access to healthcare may occur together. Developing countries carry a disproportionate burden of pesticide-related illness and death, partly due to unsafe application practices, limited awareness, inadequate protective measures, and weaker regulation of pesticide imports, exports, and use [5,10,68].

More hazardous pesticides may remain available in some low- and middle-income countries because of weaker regulatory systems, market demand, affordability, and limited enforcement [5,67]. Farmers may use older, cheaper, or more toxic pesticides because safer alternatives are less accessible or too expensive. Informal markets, repackaged products, and poor labelling can further increase misuse and accidental exposure [65]. These conditions make it difficult for users to understand the active ingredient, toxicity level, correct dilution, or protective requirements.

Weaker oversight can also lead to unsafe storage, handling, application, and disposal. Studies from agricultural communities have reported poor use of personal protective equipment, indiscriminate disposal of empty containers, and application of leftover pesticides to other crops, often linked to limited knowledge and weak regulation [64]. Limited residue monitoring in food and water further increases the likelihood that contaminated products remain undetected in local markets and community water sources [67,68].

Fewer health protections also increase vulnerability. Agricultural workers in low-resource settings may lack occupational health services, workplace training, protective equipment, and legal protections. Women, children, informal workers, and marginalized rural populations may be especially affected because their exposures are often indirect, underrecognized, or excluded from formal occupational surveillance systems [10,12,69]. Limited access to medical care and diagnostic services means that many poisoning cases and chronic health effects remain untreated or undocumented [10,68].

Training gaps further deepen inequities. Without accessible education in local languages and culturally appropriate formats, farmers and households may not understand label instructions, restricted-entry intervals, safe storage methods, or disposal requirements [65,73]. Therefore, reducing pesticide-related inequities in low- and middle-income countries requires stronger regulation, affordable safer alternatives, improved surveillance, better access to poison treatment and healthcare, community biomonitoring, residue monitoring, farmer training, and practical support for integrated pest management [5,8,67,72].

## 6. Vulnerable Populations Within Exposed Communities

### 6.1. Children

Generally, children are especially vulnerable to pesticide exposure due to various reasons, including i.e., increased dermal absorption because of a higher body surface area/weight ratio, increased respiratory rate, consumption of more food and water per body weight than adults, immature metabolic mechanisms, and habits and normal stages of development of the children [12].

Children can be exposed to multiple pesticides by multiple pathways in their schools, homes, parks, gardens, or day-care centers [76]. Their exposure is unique and can occur through dermal contact, ingestion, or inhalation pathways [77]. Direct ingestion involving swallowing pesticides, consuming contaminated food, and hand-to-mouth behavior of early childhood development are different exposure methods in children under 5 years of age [78]. Proximity of homes to pesticide-treated areas and parental occupation are reasons for organophosphate–pesticide exposure in children [16]. Children face higher pesticide exposure in the home compared to adults due to behavioral and physiological factors [79]. Residential factors such as application of insecticides and rodenticides in the home, fungicide, and herbicide usage on lawns and indoors, take-home pesticides from the workplace, proximity to treated fields, and indoor and outdoor pesticide-use patterns based on the spending time children, including school, relative homes, and child care [77]. Children may also be exposed through contact with domestic animals treated with pesticides [12].

Blewett & Nicol [43] reported that higher pesticide exposure levels were found among farm children than non-farm children, especially when present during pesticide application. Due to the exploratory behavioral factors such as crawling across the floor and playing on are increasing, the pesticide residues in the air and surfaces such as carpet, toys, and house dust are increasing through dermal, oral, and inhalation [77]. They spend more time on floors where pesticide residues may settle, frequently engage in hand-to-mouth activities and object-mouthing, have higher inhalation rates and consume more food and water per unit body weight [12,79]. Nonintentional ingestion of pesticides, including consumption of contaminated food, water, or improperly stored products, is a major cause of acute poisoning in children [12]. The 1993 National Research Council’s report highlighted the increased sensitivity of infants and children to pesticide exposure due to the pesticides in their diets. Hence, it contributed to the development of the Food Quality Protection Act of 1996, which emphasized regulatory protection against the cumulative toxicity of pesticide mixtures [47].

### 6.2. Pregnant Women and Fetuses

Pregnant women and developing fetuses represent a particularly vulnerable population to the hazards of pesticide exposure [26]. Increasing attention has been given to prenatal exposure because pesticides and other environmental chemicals may interfere with fetal development and lead to structural and functional abnormalities [80]. During pregnancy, pesticides and their metabolites may cross the placenta, resulting in direct fetal exposure, while postnatal exposure may also occur through contaminated breast milk, contributing to early-life exposure [12].

Prenatal pesticide exposure has been associated not only with adverse birth outcomes but also with neurodevelopmental effects. Epidemiological evidence has linked organophosphate exposure during pregnancy with impaired cognitive development, behavioral problems, and neurodevelopmental disorders in children, including attention and learning deficits [14]. In addition, cohort studies investigating herbicide exposure during pregnancy are emerging; although some findings remain preliminary, associations have been reported between prenatal exposure to herbicides such as glyphosate, dicamba and 2,4-D, and adverse developmental outcomes [81,82].

Household and environmental pesticide exposure during pregnancy has also been associated with adverse birth outcomes. These include preterm birth, low birth weight, and small-for-gestational-age infants, which are important contributors to infant morbidity and mortality [83]. Studies in agricultural populations have reported elevated levels of dialkylphosphate (DAP) metabolites in pregnant women exposed to organophosphate pesticides during pregnancy and the immediate postpartum period [12]. Dichlorodiphenyltrichloroethane (DDT), a persistent organochlorine pesticide with endocrine-disrupting properties, has been associated with increased risks of preterm birth and small-for-gestational-age infants [83].

Biomonitoring studies in agricultural regions have also confirmed fetal exposure to pesticides, with pesticide residues detected in maternal urine and amniotic fluid [84]. Prenatal pesticide exposure may disrupt hormonal regulation and normal developmental processes, contributing to pregnancy complications and adverse reproductive outcomes, including miscarriage and stillbirth [57,85].

### 6.3. Women in Agricultural Communities

Although males are the primary exposure in occupational pathways, women’s exposure is also considerable because of a substantial number of women workers in the agricultural sector globally [26]. Women who work both commercially and in small-scale farming are exposed to pesticides through multiple routes and modes [10]. They can be directly exposed to pesticides as applicators, and, indirectly, they are exposed as household managers and farm workers [86]. Medithi [19] reported that in the Iowa farm family exposure study, spouses who were present during pesticide application by their husbands showed about 1.5 times higher urinary biomarker level compared to women who were not present, although the differences were not statistically significant. London et al. [69] reported several pathways of women’s exposure to pesticides in the agricultural community, including mixing of pesticides, application of pesticides, aerial spray maker, reusing pesticide containers, washing contaminated clothes, contacting pesticide residues during field work, such as thinning, weeding, harvesting, and agricultural drifting, and contacting contaminated fodder. Even though women do not apply pesticides directly, they are highly exposed to some activities such as mixing chemical solutions for tractor sprayers or backpack sprayers used by men [69]. In addition, since women are mainly concentrated in higher pesticide-applied agricultural sectors such as fruit growing and floriculture, the indirect pesticide exposure is multiplied [69]. In rural areas, reusing pesticide containers for storage, cooking, or washing is another significant exposure pathway at catastrophic levels [69]. One previous study showed that women were highly exposed to pesticides compared to men in the rural agricultural community in Kenya [10].

### 6.4. Elderly Individuals

Older adults are particularly vulnerable to pesticide exposure because age-related changes in physical and cognitive function can increase both exposure and the risk of adverse health effects [87]. General activities such as frequent application of sprays in the kitchen without proper cleaning or ventilation increase both direct and indirect exposure risks [58]. In particular, the association between pesticide exposure and adverse health effects is increasing among older adults with a low level of a healthy diet [87]. In addition, elderly people use pesticides in outdoor areas where they spend more time, thereby increasing pesticide exposure [58]. Household pesticide exposure is associated with increased risk of mortality, particularly from cardiovascular disease in elderly individuals. Previous studies reported that increased urinary metabolite levels related to household insect repellents were observed, suggesting higher internal exposure in older adults [87].

### 6.5. Individuals with Chronic Illness

People with chronic diseases often experience increased harm from pesticide exposure. Some studies reported that pre-existing respiratory diseases, including chronic obstructive pulmonary disease (COPD) and asthma, can create adverse respiratory effects [59]. Previous studies showed that children with asthma who live in low-income, multi-family housing are more susceptible to adverse health concerns from pesticide exposure. In addition, they are more vulnerable to pesticide exposure due to the persistent pest infestation [88]. Respiratory symptoms such as mucus hypersecretion and bronchospasm (airway smooth muscle contraction) are caused by the inhibition of acetylcholinesterase by organophosphate and carbamate pesticides. These effects can cause severe responses in breathlessness, cough, and wheezing, especially in individuals with pre-existing conditions such as asthma or COPD [59]. Abnormal glucose regulation (AGR) (diabetes and pre-diabetes) is an adverse health effect associated with pesticide exposure. A cohort study showed that pesticide exposure moderately increased the risk of developing AGR among diabetic populations [60]. Santos-Lobato & Schuh [61] showed that heavy household pesticide application is associated with faster clinical progression of Parkinson’s disease, particularly cognitive symptoms in Parkinson’s patients. In addition, cardiovascular studies found that workers reporting pesticide exposure increase the risk factor of the prevalence of coronary heart disease [89].

### 6.6. Marginalized and Underserved Rural Populations

Rural, marginalized populations are more vulnerable to pesticide exposure, and it has become a big burden to them. These communities often lack reliable information, training, clean water, healthcare, and have minimal political voice [90,91]. Rural areas are identified as the most pesticide-exposed in the urban–rural distinction due to the presence of more plantation fields and proximity to pesticide application sites [26]. Martin et al. [92] reported that marginalized communities have to face pesticide exposure problems arising from contaminated groundwater, which are worsening due to a lack of resources and remediation assistance. In addition, developing countries shoulder a disproportionate burden of pesticide exposure and its adverse health effects, where most of the annual deaths and chronic illnesses occur. The major reasons for this issue are improper pesticide application practices, limited awareness, and a lack of protective measures. The lack of strict laws and regulations for pesticide exports and imports also contributes to this fact [5]. Mergia et al. [64] showed that a higher proportion of farmers in Ethiopia do not use personal protective equipment when handling pesticides, dispose of empty pesticide containers indiscriminately, and apply the leftover pesticides to other crops due to the poor knowledge regarding pesticides.

Several population groups are disproportionately affected by pesticide exposure due to physiological, occupational, social, and environmental factors. These vulnerable groups and their major health concerns are summarized in Table 2.

## 7. Community-Level Health Effects of Pesticide Use

### 7.1. Acute Health Effects

#### 7.1.1. Acute Poisoning Syndromes

Acute pesticide poisoning is a major global public health concern both for children and adults [78,94]. It is estimated that around 20,000 deaths result from acute poisoning annually [12]. After exposure to pesticides, within minutes to several hours, the acute toxic effects can develop [5]. The direct contact of pesticides applied to crops affects the skin, mouth, eyes, and respiratory tract [2]. A cholinergic crisis causes acute reactions, including vomiting, nausea, headache, diarrhoea, abdominal cramps, sneezing, irritation, urinary incontinence, salivation, miosis, dizziness, muscle paralysis, lacrimation, bradycardia, bronchorrhea, fasciculations, confusion, coma, seizures, hypotension, respiratory failure, and skin rashes can be caused [2,5]. Pesticide-exposed farmworkers widely experienced acute health symptoms such as headaches, nausea, and respiratory problems [4]. In Asian and African countries, the pesticide poisoning incidence rate varied from 1.1 to 1.6 per 100,000 children [12]. Healthy people who are repeatedly exposed to pesticides increase the development of respiratory conditions such as COPD and asthma [59]. The study of Ngowi et al. [70] reported that dizziness, dermal effects, and headache were the most commonly reported acute symptoms, while nausea and stomachache were less commonly reported.

Exposure to highly toxic pesticides can lead to severe acute health effects in children, including neurological and respiratory complications [12]. These effects may occur because some pesticides affect peripheral muscarinic and nicotinic receptors, as well as the central nervous system [5,77]. Acute exposure to pesticides in children showed different clinical effects based on the exposure route, pesticide type, dose, and duration of exposure [12]. Early symptoms include flu-like features and hypersecretion, followed by miosis and cardiovascular changes. Disease progression may result in neuromuscular and respiratory impairment [77]. For instance, upper and lower respiratory tract symptoms, dermal or ocular irritation, allergic responses, asthma, gastrointestinal symptoms, neurological sequelae of acute poisoning, and neurological symptoms can be shown as acute poisoning syndromes in children who are exposed to pesticides [12]. Lekei et al. [78] reported that accidents and suicides are the most common pesticide poisoning among children, while 16–17-year-old children and the female community are the most vulnerable group. Acute poisoning can result in significant psychological trauma among survivors. For instance, a study of smallholder farmers showed that they experienced acute pesticide poisoning at least one episode during their lifetime. In addition, it reported that self-reported acute pesticide poisoning was associated with neurological symptoms, including fainting, hand tremors, and increased irritability and anger. It suggests that possible psychological distress after exposure [94].

#### 7.1.2. Acute Household and Neighborhood Exposure Events

Acute pesticide exposure is not limited to occupational pathways but also occurs through household and neighborhood-level events that are often underreported. Spray drift and volatilization are important pathways through which pesticides disperse beyond the treated areas. It leads to exposure among non-farmworker residents living in proximity to agricultural lands [25,74]. Alarcon et al. [74] reported that pesticide exposure among students and school employees was due to the drift exposure from neighbouring farms. In addition to drift-related exposure, acute pesticide exposure may occur through contamination of groundwater and surface water. Agricultural runoff and poor management practices can contaminate drinking water sources. It creates a significant risk to communities relying on these sources for drinking water, contributing to the global burden of acute exposure [2]. Curl et al. [93] discussed that in the agricultural spray season, urinary glyphosate concentration of pregnant women is increased compared to the non-spray season. It suggested that agricultural glyphosate spray is an important source of exposure for people residing near fields [93]. Furthermore, unsafe storage and handling practices and sprayed pesticide residue within and around houses can lead to accidental exposure among children through contact with contaminated materials or acute non-intentional ingestion [12]. The World Health Organization (WHO) reports that approximately 1 million cases of acute poisoning occur globally [2]. Although these acute exposure events occur, many cases remain unrecognized or are not classified as pesticide poisoning. It leads to a significant underestimation of the true burden of pesticide-related acute health effects in vulnerable communities.

### 7.2. Chronic Non-Communicable Health Effects

Long-term exposure to pesticides causes several chronic diseases in humans due to an increase in toxin concentration inside the body organs, including cancer, neurotoxicity, reproductive disorders, cardiac disease, asthma, diabetes, DNA damage, impaired immune function, allergic sensitization, behavioral diseases, congenital malformation, premature death, and respiratory disorders [2,5,71,95].

#### 7.2.1. Cancer-Related Outcomes

Cancer is the second-most deadly disease worldwide, while pesticide exposure, particularly the occupational pathways, has been identified as a potential risk factor for cancer development [95]. Epidemiological and experimental studies provide evidence for carcinogenic effects of pesticides [31]. Several pesticide groups and individual compounds, including organochlorine insecticides, triazines, DDT, atrazine, diazinon, malathion, and γ-HCH, have been associated with increased cancer-related risks in epidemiological and toxicological studies [11]. It is important to note that pesticide toxicity is not limited to active ingredients alone. Commercial pesticide formulations contain so-called “inert” or co-formulant substances, many of which may also contribute to toxicity. In some cases, formulated products have been shown to exhibit higher toxicity than the active ingredients alone, highlighting the importance of considering the full formulation in risk assessment [96]. Reported cancer outcomes include non-Hodgkin’s lymphoma, leukemia, multiple myeloma, soft-tissue sarcoma, and cancers of the brain, colon, pancreas, lung, bladder, lip, and stomach [11,16,31,85,97]. Previous studies have reported that Hodgkin’s disease, melanoma, prostate, and testis cancers are particularly prevalent in men, while sinonasal, breast, ovary, and cervical cancers are particularly prevalent in women due to pesticide exposure [11]. In addition to adult cancers, concerns have also increased regarding the susceptibility of children to pesticide-related carcinogenic effects. Although evidence remains limited, a growing body of research suggests that pesticide exposure may increase the risk of childhood cancers, particularly leukemia, non-Hodgkin lymphoma, and brain tumors [12,77]. Meta-analyses and systematic reviews have shown a significant relationship between residential pesticide exposure and childhood leukemia. The evidence is stronger for indoor insecticide application, maternal exposure during pregnancy, and other prenatal or household exposure pathways [12].

#### 7.2.2. Neurological and Neurobehavioral Outcomes

The nervous system is highly vulnerable to several chemical classes in pesticides, causing long-term neurobehavioral deficits, abnormalities in nerve function, and depression [16,31]. High-level exposure to most types of pesticides, including carbamates, organophosphates, fumigants, and fungicides, can cause neurotoxicity and affect central and peripheral nervous system [16]. Pesticide poisoning is known to cause both acute and chronic neurotoxic syndromes, while long-term pesticide effects on the nervous system cause adverse effects, such as cognitive impairment, neurodegenerative, and neurodevelopmental disorders and psychomotor dysfunction [98]. Acute neurotoxicity is induced by several pesticides, including carbamates, organophosphates, organochlorine insecticides, and pyrethroids [99]. Organophosphate (OP) neurotoxic responses were studied in detail. The less severe OP poisoning neurotoxic responses show symptoms such as dizziness, headache, vomiting, nausea, pupillary constriction, excessive sweating, salivation, and tearing. More severe OP poisoning cases show muscle weakness, muscle twitches, bronchospasm, and changes in heart rate, and they can progress to convulsions and coma [16,100]. Some studies showed that pyrethroid pesticides can cause a higher risk of difficulties in their neurobehavioral, neurocognitive, or neuromotor performance in agricultural workers and/or their children [101].

Kori et al. [102] reported that pesticide exposure is associated with neurobehavioral cognitive effects, including memory impairment, reduced attention, reduced alertness, learning difficulties, and cognitive deficits. Workers who were exposed to DDT or OPs reported several moods and effects, including higher levels of depression, anger, irritability, aggressive behavior, mood disturbances, and tension [16,102]. Some studies showed that pesticide exposure is associated with mental and emotional effects, including psychiatric morbidity, suicide, depression, death from mental disorders, and neurotic disorders, particularly in women [98]. Neurodegenerative diseases, including Alzheimer’s disease, Parkinson’s disease, and multiple sclerosis, are examples of very chronic nervous disorders caused by pesticide exposure [102,103]. Previous reports showed that longer duration of pesticide exposure progressively increased the risk of Parkinson’s disease. For instance, 5 to 10 years of exposure was linked to 5% and 11% higher risks of Parkinson’s disease, respectively [104].

Effects of pesticide exposure on the developing fetus and child are a major concern for society and regulators. High-level prenatal and early childhood exposure causes neurodevelopmental outcomes, including autism spectrum disorder, ADHD, reduced intelligence, developmental delays, learning disabilities, emotional behaviors, slower response speed, visual motor and visual spatial deficits, and impaired motor deficits [105,106,107]. These findings highlight pesticide exposure as an important environmental risk factor for child neurodevelopment.

#### 7.2.3. Respiratory Outcomes

Pesticide exposure is related to various respiratory issues, particularly in farm workers, including chronic lung diseases, chronic bronchitis, chronic obstructive pulmonary disease (COPD), sarcoidosis, rhinitis, organic dust toxic syndrome (ODTS), and asthma [1,59,71]. The common respiratory symptoms associated with pesticide exposure are airway irritation, wheezing, cough, dry/sore throat, breathlessness, and chest tightness [1]. Direct exposure to pesticides, mostly spray drift inhalation and dermal exposure, raises concerns for the operator regarding respiratory issues. Pesticides have the possibility of causing acute irritant responses in the lung [59].

Occupational pesticide exposure has been associated with impaired respiratory health, including reduced lung function, chronic bronchitis, and increased risk of chronic obstructive pulmonary disease (COPD) [108]. Evidence from agricultural and pesticide-industry workers suggests that men may have a higher incidence of COPD following occupational pesticide exposure. In addition, non-smoking female farmers have shown increased risks of chronic bronchitis and other respiratory symptoms associated with the use of several pesticide groups and compounds, including organochlorines such as DDT and toxaphene, organophosphates, carbamates, and permethrin [1].

Pesticide exposure has also been associated with asthma-related outcomes. Pathak et al. [2] reported that several application methods, including aerosol use, indoor spraying, and over-the-counter insecticide application, may aggravate asthma among non-farm workers. Reported respiratory symptoms include wheezing, airway irritation, dyspnea, dry cough, lower respiratory discomfort, shortness of breath, and chest tightness [2]. Occupational pesticide exposure has also been linked to adult-onset asthma, allergic asthma, occupational asthma, asthma exacerbation, and wheezing, with some studies reporting dose-dependent associations [1]. Some evidence also suggests that these associations may be stronger in women than in men [1]. In addition, prenatal and early-childhood exposure may influence immune-system development and increase later asthma risk, particularly through residential and take-home exposure pathways [77].

#### 7.2.4. Reproductive and Developmental Outcomes

Pesticide exposure has been associated with adverse reproductive and developmental outcomes in both men and women [11,85]. In women, exposure has been linked to fetal death, early pregnancy loss, infertility, early menopause, spontaneous abortion, intrauterine growth restriction, and congenital malformations [11]. These effects may vary according to sex, age, diet, occupation, exposure level, and pesticide type. Some reproductive outcomes may be partly related to endocrine disruption and altered female hormonal function [104]. Several studies have also reported an association between pesticide exposure and longer time to pregnancy, suggesting possible impairment of reproductive capacity [98]. Fetuses, infants, and children are more vulnerable to pesticide toxicity than adults [104]. Pesticide exposure has been significantly associated with an increased risk of birth defects. Specific birth defects linked to pesticide exposure include urogenital anomalies, orofacial clefts, limb reductions, central nervous system defects, eye anomalies, and heart defects [98]. Albadrani et al. [109] showed that there is a higher percentage (41%) of spontaneous abortion risk in women exposed to pesticides.

Some organochlorine pesticides such as DDT, DDE, lindane, and dieldrin have been shown to interfere with androgen synthesis and action, which cause impairing normal reproductive development [85]. Roberts et al. [77] indicated that DDT is the major organochlorine that associated with fetal death and birth defects. Fetal exposure is occurring via maternal serum or umbilical cord blood levels, which are associated with preterm birth, intrauterine growth retardation and decreased birth weight. There is considerable concern regarding the physical development of the embryo and fetus from pesticide exposure [77]. Reported reproductive issues in males include sperm abnormalities, sperm chromosomal defects, delayed puberty, reduced semen quality, decreased sperm count, endocrine disruption, infertility, and erectile dysfunction [85,98]. Epidemiological studies reported that pesticides such as lufenuron, novaluron, fipronil, bifenthrin, and temephos are contributing to male infertility by reducing sperm capacitation, motion parameters, sperm motility, and sperm cell viability [110].

#### 7.2.5. Endocrine, Metabolic, and Cardiovascular Outcomes

Many pesticides have been identified as endocrine-disrupting chemicals (EDCs) because they interfere with natural hormones and mimic the action of the natural hormone. These EDCs have the possibility to interfere with the hormone synthesis, transportation, metabolism, and elimination [111]. According to the evidence, EDCs may cause alterations in pubertal development and thyroid function, which may increase susceptibility to obesity [112]. Organochlorine pesticides, including DDT, methoxychlor, chlordane, dieldrin, endosulfan, and some fungicides and herbicides, have been reported as having an association with negative effect of endocrine system in humans [77]. For example, some endocrine disruptor pesticides, such as amitrole, zineb, maneb, mancozeb, fipronil, cyhalothrin, ziram, and ioxynil, can inhibit thyroid hormone production [111]. Endocrine disruptors can cause a variety of adverse effects, such as autism spectrum, delay developments in children after prenatal exposure, impaired learning, memory, behavior, attention, depression, anxiety, and sensation in children [112].

Several epidemiological studies have highlighted that pesticide exposure, particularly organophosphate pesticides, is associated with a higher incidence of diabetes [71,113]. Many pesticides act as EDCs, which may cause hormonal imbalances and disrupt glucose metabolism, thereby increasing the risk of diabetes [113]. The potential mechanisms by which pesticides disrupt glucose homeostasis and impair glucose regulation include inhibition of acetylcholinesterase activity, reduced insulin secretion, pancreatic beta-cell dysfunction, lipotoxicity, oxidative stress, inflammation, gut microbiota dysbiosis, and endocrine disruption [113,114]. Some evidence suggests that women may be more susceptible to pesticide-related diabetes, especially in the agricultural sector. In addition, prolonged exposure to certain pesticides, such as organochlorine and organophosphate insecticides, frequent pesticide spraying, inadequate use of personal protective equipment, and exposure to multiple pesticide mixtures further increase the risk of type II diabetes [113,115].

In addition, pesticide exposure can affect the cardiac health of humans by resulting in cardiac abnormalities and death in severe cases. For instance, CPF, dichlorvos, and parathion, as organophosphate pesticides, may be linked to various cardiovascular complications, including prolonged parasympathetic activity, electrocardiographic abnormalities, a transient increase in sympathetic tone, and myocardial damage [110]. Zago et al. [116] showed that fenitrothion, primaphos, deltamethrin, and malathion pesticides are associated with increasing blood pressure. A cohort analysis showed that Japanese-American males who are highly exposed to pesticides by occupational pathways correlated with cardiovascular disease incidence over 10 years [117].

#### 7.2.6. Dermatological, Immunological, and Allergic Outcomes

Dermal contact is one of the main routes of pesticide exposure, particularly among workers who handle, mix, or apply pesticides [95]. Such exposure can lead to irritant or allergic skin reactions, with irritant contact dermatitis and allergic contact dermatitis being among the most commonly reported pesticide-related skin conditions. Less common dermatological outcomes associated with pesticide exposure include erythema multiforme, contact urticaria, occupational acne, hair and nail disorders, skin cancer, porphyria cutanea tarda, and ashy dermatosis [118]. Although pesticide sprayers are mostly prone to dermatological effects, farmers are also exposed to pesticides during mixing, loading, cleaning equipment, and disposing of empty containers [118,119]. Among farmers involved in DDT spraying, pesticide-related hair loss has been reported and classified as diffuse alopecia of mixed type. Contact dermatitis has also been observed among pesticide sprayers following exposure to the carbamate herbicide Carbyne, which was subsequently accompanied by hypopigmentation of the affected skin area [118]. Several studies have reported a dose–response relationship between dermatitis and poor application practices or prolonged exposure duration [98]. In addition, many daily products, such as medications, rubber, and household cleaning products, may contain pesticides or chemically related compounds capable of triggering relapses of contact dermatitis [118]. Recent evidence from Asia suggests that improper application of personal protective equipment and unsafe handling practices remain a major contribution to pesticide-related skin and eye symptoms [120]. Pesticide sprayers and other people who are exposed to herbicides and insecticides are also prone to nail dystrophy, characterized by nail discoloration, deformities, and eventual nail loss [118].

Exposure to pesticide-related compounds through water, air, or food has been associated with increased risk of allergic disorders, such as asthma, allergic rhinitis, and atopic dermatitis [2,121]. Certain chemicals, such as dichlorophenols, may kill the human microbiota, which potentially increases allergy susceptibility during childhood [2]. Epidemiological studies reported that children with high urinary dichlorophenol levels were associated with an 80% increased likelihood of food allergy [2]. Certain estrogenic pesticides may promote allergic reactions by stimulating mast cell release of allergic mediators [122].

Pesticides can directly damage immune cells and lymphoid tissues by leading to immunotoxicity. This may damage immune cell development, survival, signaling, and function [122]. Because immune responses are strongly regulated by hormones, disruption of endocrine pathways by pesticides may lead to immune dysregulation [122]. Faustini et al. [123] showed that short-term immunosuppressive effects can occur due to agricultural exposure to commercial 4-chloro-2-methylphenoxyacetic acid (MCPA) and 2,4-dichlorophenoxyacetic acid. Pesticide exposure has been associated with autoimmune dysfunction through mechanisms such as molecular mimicry, altered self-antigen recognition, and endocrine disruption, which may trigger immune responses against self-tissues. Epidemiological and case-based studies have suggested that exposure to some pesticides, including chlorpyrifos, glyphosate, organochlorines, and malathion, may be associated with immune dysregulation and increased autoantibody levels [123]. Pesticide exposure has also been discussed in relation to autoimmune diseases such as rheumatoid arthritis, pemphigus, systemic lupus erythematosus, and myasthenia gravis [123].

### 7.3. Subclinical and Biomarker-Based Effects

Pesticide exposure has been associated with oxidative stress, DNA damage, and other genotoxic effects, even at low or sublethal exposure levels [124]. Biomarkers can help detect early biological changes in exposed populations, but they should be interpreted according to their specific meaning. For example, DNA damage markers indicate genotoxic effects, whereas acetylcholinesterase inhibition is mainly used as a biomarker of exposure or biological effect for cholinesterase-inhibiting pesticides, such as organophosphates and carbamates [124]. These biomarkers may be useful for identifying early community-level health risks before clinical disease develops.

Genotoxic effects are important because they may represent early indicators of long-term health risks, including cancer and adverse reproductive outcomes [56]. Urinary metabolites of pyrethroid pesticides have been associated with sperm DNA fragmentation, reduced semen quality, and chromosomal abnormalities, suggesting possible reproductive toxicity [2]. Genotoxicity refers to the ability of a substance to damage genetic material within cells. Epidemiological studies have reported associations between exposure and some pesticide groups, including organophosphates and pyrethroids, and increased chromosomal aberrations, which may be linked to reproductive effects, cancer risk, and developmental outcomes [98]. Non-occupational exposure among people living near agricultural land has also been associated with higher pesticide exposure and increased DNA damage [2].

Pesticide exposure may produce subclinical biological effects that can be detected through biomarker-based monitoring. Cytogenetic biomarkers such as chromosomal aberrations, micronuclei frequency, single-cell gel electrophoresis, and sister chromatid exchanges have been widely used to analyze genetic damage in pesticide-exposed populations [56,125]. The magnitude of biomarker alterations may differ according to occupational pesticides, including personal protective equipment use and work environment. In addition, biomarkers such as pesticide metabolites, hemoglobin, or albumin adducts, DNA adducts and oxidative stress markers can reflect internal dose and early cellular injury [56]. Increased micronuclei frequency, oxidative damage, and DNA strand breaks have been reported in toddlers living in pesticide-sprayed areas by highlighting early-life vulnerability [2]. Recent biomonitoring evidence from children living in agricultural communities in Mexico showed that chronic pesticide exposure was associated with significantly shorter telomere length. Telomeres are specific DNA and protein complexes located at the ends of chromosomes. It suggested accelerated biological ageing and the development of genomic stability [126]. Although these markers do not always predict specific diseases, they are valuable tools for identifying early biological effects and estimating community health risks before clinical symptoms become visible [56].

## 8. Beyond Disease: Broader Community Consequences

### 8.1. Household Economic Burden

Exceptional and continuous pesticide application imposes costs on farmers and society [62]. The household economic burden associated with pesticide exposure occurs due to several reasons, including medical treatment, hospitalization expenses, inability to work due to temporary disability, reduced future earnings due to long-term health effects after poisoning, caregiving costs when family members append time caring for poisoned children, funeral expenses in fatal poisoning cases, lost time for the mourners, and extra costs for prevention methods such as safer pesticides and personal protective equipment [127]. Despite the agronomic benefits of pesticide application, substantial household-level costs may arise, including pesticide purchase expenses, healthcare costs related to acute and chronic adverse effects, and spending on personal protective equipment such as gloves and protective clothing [62,66].

Because farmer health impacts are frequently underreported in national pesticide statistics, the true household economic burden of pesticide dependence is sometimes overly underestimated [128]. Pesticide exposure in people results in loss of productivity and wages, as well as increased medical expenses, due to increased risk of adverse health issues, leading to a decline in economic loss and human productivity, which marginalizes for poor farmers [62]. Cole & Leon [127] reported that a higher rate of acute poisoning in Ecuador is due to occupational exposure and the significant economic burden, with costs exceeding five times the daily agricultural wage. Previous studies estimated that the total cost of pesticide use and exposure is responsible for approximately 15% of the household agricultural income and 5% of gross household income. The burden is particularly higher among small-scale farmers [62]. However, the household economic burden is likely underestimated for several reasons, including underreporting because many poisoning cases do not seek healthcare, limited awareness of pesticide poisoning symptoms, long-term complications that are not fully identified or measured, overlooked indirect household costs, and lost earnings due to deaths that are not fully accounted for [127].

### 8.2. Educational and Developmental Consequences

Beyond financial consequences, pesticide exposure may also be associated with educational and developmental impacts in children. Evidence suggests that exposure may affect memory, learning ability, attention, motor skills, visual memory, visuospatial performance, and overall neurodevelopment [69,102]. Children living near pesticide application areas or whose parents work in agriculture have shown poorer memory and learning outcomes during childhood and adolescence [129]. Prenatal and childhood exposure to organophosphate pesticides has also been associated with impaired neurobehavioral development [130]. Similarly, exposure to organochlorine pesticides in newborns and infants has been linked to poorer motor and cognitive development [102].

Prenatal pesticide exposure has also been associated with reduced birth weight, shorter height, and congenital malformations [12,77]. Some studies have reported associations between parental occupational pesticide exposure and a higher risk of autism spectrum disorder in children [102]. Exposure to certain pesticide groups, including organochlorines, organophosphates, and pyrethroids, has been linked to neurodevelopmental disorders such as autism spectrum disorder, cognitive impairment, and attention-deficit/hyperactivity disorder [131]. Chronic pesticide exposure may also be associated with learning and memory impairment, slower reaction time, poorer short-term memory, reduced attention, lower alertness, and decreased cognitive performance [69,102].

### 8.3. Physical and Mental Health Impacts

Chronic pesticide exposure is associated with significant mental and physical health risks, including neurological disorders and endocrine disruptions, particularly in people with high exposure settings like agriculture [132]. Depression, irritability, heightened anxiety, impulsivity, obsessive compulsiveness, suicidal tendency, and behavioral alterations are some mental health impacts associated with pesticide exposure [102,112]. Epidemiological studies indicate that there are the highest rates of poor mental health and diagnosed mental health disorders and suicide rates among farmers compared with some other occupations. For instance, Australian agricultural workers were reported to have the highest suicide rates, which is twice that of other occupations. Reasons for these suicidal incidents included financial, interpersonal, or work-related stress [133]. Ong-artborirak et al. [134] reported that farmers had significantly higher rates of anxiety/insomnia and severe depression than controls. This elevated risk of depression, impulsivity, suicide ideation, disturbance in moods, and anxiety was associated with exposure to organochlorines, organophosphates, pyrethroids, herbicides, and carbamates [61]. Consumer concern about pesticide risk increases when residues are detected in food or drinking water. Some consumers may perceive any pesticide residue in food as risky, even when concentrations are below acceptable regulatory limits [35,135]. In addition, many pesticide users have limited knowledge of the health and environmental risks associated with pesticide application [136]. Such risk perception, uncertainty, and limited awareness may contribute to psychological stress and mental health burdens, particularly among farmers and communities that depend on pesticide-intensive agriculture. Beyond these concerns, pesticide exposure has also been associated with adverse mental health outcomes, including depression, and some epidemiological studies have reported an association between pesticide exposure and increased risk of suicidal behavior and suicide [137].

### 8.4. Food Security and Livelihood Implications

According to the United Nations’ Food and Agriculture Organization, most chemicals used in agriculture do not meet international safety standards and are, in fact, highly toxic to humans and the environment [138]. High dependence on pesticides is a major global concern because it may contribute to environmental contamination, food safety risks, and adverse public health outcomes. Continued pesticide application is driven by several factors, including increasing returns on investment, high costs of switching to alternative practices, market dependence, and uncertainty [63]. The “pesticide treadmill” concept explains the gradual development of pest resistance to pesticides, which pushes farmers toward the use of newer and often more toxic chemicals. Although pesticide application may initially lead to significant yield improvements, over time, yields tend to stop improving and crop prices may fall. Farmers who do not use pesticides may face financial pressure and risk bankruptcy due to reduced competitiveness. Therefore, over time, pesticide use in agricultural products has become a normal and necessary part of farming and farmers’ livelihoods [63].

Conventional modern agriculture depends heavily on chemical inputs, and these agrochemicals have contributed to the rapid growth of global food production and consumption [139]. In the general population, food is the primary source of exposure to pesticides. These pesticide residues can be found in food, including fruits, vegetables, grains, and fish, although most are within legal limits [77]. Despite offering greater benefits, fruits and vegetables significantly contribute to pesticide residue exposure in modern households [140].

### 8.5. Intergenerational Consequences

Pesticide exposure may have important intergenerational consequences, particularly for pregnant women, fetuses, and children. During pregnancy, pesticides and their metabolites may cross the placenta and reach the developing fetus. Such prenatal exposure may interfere with fetal growth, endocrine function, and normal developmental processes, and it has been associated with adverse outcomes such as preterm birth, low birth weight, congenital abnormalities, and stillbirth [12,57,83]. These effects are important because disruption during critical periods of fetal development may increase susceptibility to health problems later in life.

In childhood, pesticide exposure has been associated with neurodevelopmental impairments, including reduced cognitive function, attention deficits, behavioral disorders such as attention-deficit/hyperactivity disorder and autism spectrum disorder, and learning difficulties [102,130,131]. Children are especially vulnerable because of their developing organs, higher intake of food and water per body weight, hand-to-mouth behavior, and immature detoxification systems. Therefore, early-life pesticide exposure may have long-term consequences for education, productivity, and overall well-being, contributing to broader social and health burdens across generations.

These intergenerational impacts are not evenly distributed and are strongly shaped by environmental justice considerations. Evidence from the global literature indicates that pesticide exposure burdens are disproportionately higher in low- and middle-income countries, where weaker regulatory enforcement, limited protective infrastructure, and intensive agricultural practices contribute to elevated risk [35,141,142]. In high-income countries, similar disparities are observed at the intra-national level, where marginalized and minority communities are more likely to reside in proximity to agricultural fields or in occupational settings with higher pesticide exposure [142,143]. In the United States, studies have shown that Latino and African-American populations, particularly farmworkers and their families, experience significantly higher exposure levels compared with White populations, reflecting structural inequalities in labor, housing, and environmental regulation [144,145,146]. These patterns highlight that pesticide exposure is not only a toxicological and public health concern, but also a manifestation of broader social inequities, reinforcing the need to integrate environmental justice frameworks into pesticide risk assessment and policy development.

## 9. Challenges in Measuring Community Health Impacts

### 9.1. Difficulties in Exposure Assessment

Assessing community-level pesticide exposure remains challenging because exposure patterns are complex, variable, and often difficult to measure directly. Exposure may differ according to pesticide formulation, application method, equipment, weather conditions, use of personal protective equipment, frequency and duration of application, route of exposure, physiological factors, and contact with recently treated fields or contaminated equipment [8,31,35,57,147,148]. Communities may also be exposed through multiple overlapping routes, including dermal contact, inhalation, ingestion, residential proximity, take-home residues, and contaminated food or water.

Accurate exposure assessment is further limited by the lack of clear markers that reflect the duration, intensity, and timing of past pesticide exposure [16]. Retrospective assessment is often uncertain, while prospective measurement and repeated environmental or biological monitoring can be costly and difficult to implement [147]. As a result, many epidemiological studies rely on questionnaires, job-exposure matrices, proximity to treated fields, parental occupation, biomonitoring, or exposure models. However, each approach has limitations, including recall bias, poor historical exposure information, short biological half-lives of some pesticides, and uncertainty in model-based estimates [16,148,149,150].

These limitations can affect the interpretation of associations between pesticide exposure and health outcomes, especially for chronic diseases such as cancer, where long latency periods and mixed exposures are common [150]. Exposure assessment is particularly difficult in low-resource settings, where unauthorized pesticide use, repackaging, poor labelling, language barriers, and limited literacy may reduce the accuracy of exposure information [65]. Therefore, future community studies should combine improved questionnaires, environmental monitoring, biomonitoring and exposure modelling to better estimate cumulative, and long-term pesticide exposure [65].

### 9.2. Underreporting and Under-Ascertainment

Underreporting and under-ascertainment of pesticide-related health effects represent significant challenges in evaluating community health impacts. London et al. [10] reported that underreporting of pesticide poisoning cases is a recognized major problem in accurately measuring disease burden. Many mild or symptomatic cases are not captured by existing surveillance systems [10]. Although acute poisoning data are used as an indicator, they only reflect visible or clinical cases. Many of the exposure events are not systematically recorded [68]. Gender-related disparities further contribute to underreporting, as exposures and health outcomes of women are often underrecognized or misclassified. Sometimes, cases are dismissed as “mass hysteria” or psychological [10]. In developing countries, these limitations are exacerbated by inadequate infrastructure, lack of standardized reporting systems, and insufficient quality control in data collection [68].

There is an urgent need for valid global estimates of pesticide poisoning, as existing global estimates are outdated and likely represent substantial underestimates. Current data are largely derived from scattered case reports and small surveys, indicating incomplete surveillance coverage [31]. These limitations have important epidemiological implications because underreporting leads to systematic underestimation of exposure prevalence and disease burden, thereby weakening risk assessment, obscuring vulnerable populations, and limiting the effectiveness of public health interventions. Surveillance systems also often fail to capture dermal and chronic symptoms and non-occupational exposures, such as those occurring within households and communities [10]. As a result, many cases remain undocumented, particularly among rural populations, informal workers, and other vulnerable groups, leading to biased estimates of exposure–outcome relationships.

### 9.3. Limitations of Current Epidemiological Studies

Current epidemiological studies on pesticide exposure have several methodological limitations that affect the reliability and interpretability of findings. Study findings are often inconsistent and may be influenced by bias, mainly due to inadequate measurement of exposure or dose [147]. Variability in exposure assessment methods also contributes to inconsistencies across studies [65]. Many studies depend on self-reported data, job exposure matrices, exposure algorithms, and questionnaire-based assessments. They differ in their validity and accuracy, resulting in heterogeneous findings. They tend to recall bias and exposure misclassification [16,65,151]. In addition, cross-sectional study designs are frequently used, but they limit the ability to establish a correlation between exposure and health outcomes [8,151]. Biomonitoring of pesticides has different challenges, such as difficulty in measuring low-dose exposures, issues with short half-life pesticides, and detection limits (LOD issues) that reduce accuracy. Cross-sectional designs limit the ability to access cumulative exposure [8].

Pesticide exposure data and their health effects from epidemiological studies are cross-sectional and not longitudinal as they limit the understanding of long-term exposure effects [74]. Small sample sizes in some studies reduce statistical power and limit the ability to detect meaningful associations or to perform subgroup analyses. Additionally, weak control of confounding factors, such as age, co-exposures, socioeconomic status, and lifestyle variables, can introduce bias and obscure true exposure [151]. Harris [147] highlighted that inadequate measurement of exposure or dose is a major issue that reduces the validity of many epidemiological findings. Ockleford et al. [151] highlighted several limitations in methodologies in epidemiological studies, including bias in frequently used study designs such as case-control and cross-sectional studies, lack of prospective studies, lack of detailed exposure analyses, seldom use of appropriate biomarkers, reliance on broad definitions of exposure assessment through questionnaires, deficiencies in reporting and analysis, publication bias, and other biases and selective reporting. Further, the main limitation is uncertainty in exposure assessment. Less attention to gender-based susceptibility, inappropriate or non-validated surrogates of health outcomes and limitations in conducting repeated measurements over time are also several limitations in epidemiological studies [151].

### 9.4. Gaps in Community Biomonitoring

Despite its potential to provide direct measures of internal exposure, biomonitoring remains underutilized community-based studies of pesticide exposure. Lack of biomarkers/ indices, inconsistent ADI standards, limited LMIC data, lack of monitoring systems, no global disease, and cross-sectional biomonitoring are several reasons for gaps in community biomonitoring [67]. The application of biomarkers is limited, especially in general populations, where exposure levels are often low and may fall below analytical detection limits [8]. Biomonitoring in pesticide exposure has several limitations, such as complexity, costly analytical procedures, and requiring specialized laboratories [67]. This limitation reduces the sensitivity of biomonitoring data for detecting associations with health outcomes.

There is also a lack of routine surveillance systems for monitoring pesticide exposure at the population level. While some data are collected through poison control centers, occupational health programs and ad hoc reporting systems. These sources are normally fragmented and lack standardization [67,151]. Most pesticide biomonitoring studies are conducted in high-income countries, which creates a significant lack of data in low- and middle-income countries. However, pesticide exposure is somewhat higher in those countries, where use of personal protective equipment, regulations, and agricultural practices may be less stringent [67]. It confirms the major global biomonitoring gap.

### 9.5. Need for Integrated Community-Level Indicators

The complexity of pesticide exposure and its health effects is crucial for the development of integrated community-level indicators that combine multiple sources of information. Existing approaches emphasize the importance of incorporating environmental, biological and occupational data to improve exposure assessment. Indicators such as pesticide production and consumption data, biomonitoring results, and measurements of pesticide residues in food, water, and human tissues provide complementary information on exposure patterns [68]. Pesticide regulations differ across countries, with some having strict standards and others adopting more lenient policies. These regulatory differences are influenced by factors such as agricultural practices, pest prevalence, and regional environmental conditions. However, more lenient regulatory settings may contribute to increased population exposure in certain regions. These differences are shown in variations in pesticide application rates and application across regions [67]. Since different residue testing protocols yield varying results, research and development of a gold-standard testing protocol are needed. There is no regular monitoring of pesticide risk, which makes it difficult for legislators, regulators, farmers, and consumers to make rational decisions [152].

Biomonitoring data quantify internal exposure across all routes, providing a more comprehensive assessment of physiological burden [8]. However, these data should be interpreted alongside clinical data, such as health records, diagnostic information, and reports of acute or chronic effects. The integration of biological markers, genetic data, and mechanistic indicators such as DNA damage and oxidative stress can also enhance understanding of the presence of multiple exposures and health outcomes [56]. Given the presence of multiple exposure sources and cumulative effects, multidisciplinary approaches are required to comprehensively measure community health impacts and improve the accuracy of risk evaluation [31]. In low- and middle-income countries, the vulnerabilities to pesticide exposure, particularly for banned pesticides, are further exacerbated due to socioeconomic inequalities. Therefore, developing broad-spectrum, nonspecific biomarkers with robust analytical methods and interlaboratory harmonization are crucial to ensure data quality and comparability [67].

## 10. Public Health and Policy Responses

### 10.1. Community Education and Risk Communication

Several epidemiological studies highlighted that the awareness of farmers and agricultural workers of proper pesticide application practices and risks during pesticide exposure is low. The implementation of safe farming practices, including safe health practices of using pesticides, is hindered by several factors, such as lack of information, socio-cultural and economic barriers, and inadequate training [153]. Incorporating risk-perception studies into educational campaigns is crucial for effective community education and risk communication about pesticide exposure, using methodological triangulation and community-based participatory research methods [72]. Due to the current pesticide-induced environment, safety concerns and health hazards are critical problems. Therefore, several regulatory bodies and management approaches are involved in promoting pesticide safety and community health. Integrated Crop Management (ICM) provides guidance for reducing reliance on pesticides while supporting safe agricultural production and environmental sustainability [154]. Before applying pesticides to agricultural regions, it is critical to receive information regarding specific hazards to farmers to mitigate the risks involved in pesticide exposure. However, there are many potential issues regarding this risk communication [73]. Munoz-Quezada et al. [155] reported that community education increased risk perception but did not reduce pesticide exposure because individual-level interventions are insufficient, requiring broader regulatory measures. Some studies addressed the need for better communication campaigns and information for farmers to increase awareness of health hazards associated with uncontrolled and improper pesticide use [156,157,158]. There are several potential barriers to pesticide risk communication, including nonuniform methods of pesticide safety training and language differences [73].

Unsafe storage practices, including the reuse of pesticide containers for storing food or water, contribute to pesticide exposure among farmers. In addition, mishandling during the transfer of pesticides from original containers to household containers and failure to follow label guidelines can increase the risk of occupational exposure [19]. Previous studies reported that farmers who read labels on pesticide bottles use safer pesticide practices compared to farmers who cannot read the labels. It confirms that illiterate farmers are a highly vulnerable group to pesticides due to their inability to understand instructions regarding safety and concerns. It highlights the importance of community education toward safe pesticide practices [4].

These facts arise due to the lack of knowledge and awareness among farmers and their family members. Much of the misuse of pesticides occurs after the point of sale, leaving pesticide use largely uncontrolled. Provision of safe pesticide disposal sites in all agricultural districts is needed. Updated legislation could better control pesticide importation, sale, and use in Thailand [153]. Research demonstrates that communication through community leaders, religious leaders, and local influencers produces better educational outcomes in agricultural communities [159]. Participatory approaches in science communication were found beneficial because communities became more engaged with local leaders [160].

### 10.2. Primary Prevention Strategies

Practicing eco-friendly agriculture is essential to prevent further environmental degradation and promote the recovery of the already degraded environment [85]. Safe use practices of pesticides among farmers in developing countries are associated with several factors, including good knowledge and attitudes, having more than 5 years of experience in pesticide application, and being educated [4]. Therefore, improving the knowledge and attitude levels about the safe use of pesticides is an essential requirement through several strategies, such as training and capacity building [4,85]. Making the awareness of farmers regarding the necessity of using personal protective equipment is another important strategy to prevent pesticide exposure [85]. Application of bioremediation methods is a trending, eco-friendly approach to remove harmful toxins for making a sustainable environment [2]. Encouraging the application of integrated pest management techniques, such as the use of pheromones, light traps, bonfires, and trap crops, is an important primary prevention strategy [85]. In addition, broader agricultural transitions such as agroecology, organic farming, and regenerative agriculture can substantially reduce reliance on synthetic pesticides, thereby lowering environmental contamination and reducing human exposure and associated health risks.

FAO-supported regional harmonization initiatives included guidelines on pesticide registration, labelling, and residue monitoring as a prevention approach [161]. To reduce the pesticide residue in raw agricultural commodities, the worldwide harmonization of maximum residual limits (MRLs) has attained a high level of recognition globally. For instance, in India, the Food Safety and Standards Authority of India (FSSAI), like central agencies and food technologists, are placing increasing emphasis on a sustainable food sector to lower residual limits of pesticides in food commodities [2]. Current regulations increasingly focus on the Precautionary Principle and minimum criteria for pesticide use in accordance with international standards [153]. Epidemiological studies confirmed that peer education programs, field schools, and community workshops improved understanding of pesticide risks and promoted safer practices [162]. Research highlighted the need to protect vulnerable populations such as children and nearby communities from pesticide exposure [153].

## 11. Conclusions

Pesticide exposure should be understood as a community health problem rather than only an occupational hazard. The evidence summarized in this review shows that exposure can occur through agricultural work, take-home residues, residential proximity, food, water, air, household use, and public spaces. These pathways affect workers, families, children, pregnant women, older adults, chronically ill individuals, and marginalized rural populations. Health outcomes range from acute poisoning to chronic neurological, respiratory, reproductive, endocrine, cardiovascular, dermatological, immunological, and subclinical biomarker-based effects. Broader consequences include economic costs, educational and developmental impacts, mental health burdens, and intergenerational vulnerability. Findings from arid agricultural regions, including examples from Saudi Arabia, further highlight the importance of environmental monitoring of food products, groundwater, and agricultural soils to reduce long-term community exposure risks. Public health responses should combine safer pesticide governance, integrated pest management, community education, biomonitoring, improved surveillance, stronger protection for vulnerable populations, and locally appropriate prevention strategies.

## Figures and Tables

**Table 1:** Community-level pesticide exposure pathways, affected groups, prevention points, and supporting references.

Exposure Pathway	Primary Exposure Sources	Community Groups Mainly Affected	Key Exposure Routes	Risk Mitigation Strategies	References
Occupational exposure	Mixing, loading, spraying, harvesting in recently treated fields, equipment cleaning	Farmers, farm workers, greenhouse workers, pesticide applicators, florists, manufacturing workers	Dermal contact, inhalation, accidental ingestion	Training, correct pesticide dilution, proper PPE, safe equipment cleaning, restricted-entry intervals	[1,5,17,18,19]
Para-occupational (take-home) exposure	Contaminated clothing, shoes, hair, skin, vehicles, tools and PPE brought home	Families of agricultural workers, especially children and pregnant women	Dermal contact, dust ingestion, inhalation of residues	Washing work clothes separately, changing before entering home, storing PPE outside living areas, workplace shower facilities	[1,42,43]
Residential exposure	Household pest control, garden/lawn pesticides, nearby agricultural spraying, contaminated house dust	Rural residents, peri-urban communities, children, elderly individuals, non-farm households near fields	Inhalation, dermal contact, dust ingestion	Buffer zones, drift control, safer household pest management, regular wet cleaning, improved ventilation	[19,25,31,44]
Dietary exposure	Pesticide residues in fruits, vegetables, grains, dairy products, fish and baby food	General population, children, pregnant women, infants	Ingestion	Monitoring residues, observing pre-harvest intervals, washing/peeling food, promoting integrated pest management	[19,20,35,36,46,47]
Drinking water exposure	Agricultural runoff, leaching to groundwater, equipment washing, contaminated surface water	Rural households, communities using wells or untreated water, livestock-dependent communities	Ingestion, sometimes dermal contact	Water testing, safe disposal of containers, avoiding washing sprayers near water sources, watershed protection	[33,34,49,50,51,52]
Airborne and public-space exposure	Spray drift, volatilization, mosquito-control aerosols, pesticides used in schools, parks and public spaces	Schools, children, residents near fields, public-space users	Inhalation, dermal contact	No-spray buffer zones near schools/homes, weather-based spraying restrictions, public notification before spraying	[25,45,55]
Cumulative and mixed exposure	Combined occupational, household, dietary, water and air exposure	Agricultural communities, plantation communities, women, children, elderly people, chronically ill individuals	Multiple routes	Community biomonitoring, cumulative risk assessment, integrated health surveillance	[8,56]

**Table 2:** Vulnerable populations, exposure pathways, and supporting references.

Vulnerable Group	Why Vulnerability Is Increased	Common Exposure Settings	Heath Outcomes	Implications	References
Children	Higher food and water intake per body weight, hand-to-mouth behavior, higher respiratory rate, immature metabolic and developmental systems	Homes, schools, farms, gardens, contaminated dust, diet, treated pets	Acute poisoning, neurodevelopmental effects, asthma, learning and behavioral problems, childhood cancers	Early-life exposure may be associated with developmental, educational and long-term productivity-related outcomes	[11,12,47,76,77,78,79]
Pregnant women and fetuses	Transplacental exposure, fetal developmental sensitivity, hormonal disruption, exposure through breast milk after birth	Agricultural work, residence near fields, household pesticides, contaminated diet and water	Preterm birth, low birth weight, congenital abnormalities, miscarriage, stillbirth, developmental effects	Maternal and child health outcomes may be affected, with possible intergenerational implications	[12,57,80,83,84,85]
Women in agricultural communities	Direct farm work, mixing pesticides, washing contaminated clothing, household management roles, indirect field exposure	Fields, plantations, homes, pesticide storage areas, contaminated clothing and equipment	Reproductive effects, endocrine disruption, poisoning, chronic illness	Exposure among women may be underestimated, underreported, or misclassified in some settings	[10,19,26,69,86]
Elderly individuals	Age-related physiological changes, chronic disease burden, higher sensitivity to toxicants	Household pesticide use, gardens, rural homes, contaminated food and water	Cardiovascular disease, respiratory problems, neurological effects, increased mortality risk	Older adults may be more vulnerable because of reduced physiological resilience and higher healthcare needs	[58,87]
Individuals with chronic illness	Pre-existing respiratory, neurological, metabolic, or cardiovascular disease may increase susceptibility	Homes, workplaces, agricultural communities, contaminated indoor environments	Asthma/COPD exacerbation, diabetes-related effects, Parkinson’s disease progression, cardiovascular outcomes	Pesticide exposure may interact with existing diseases and contribute to health inequalities	[59,60,61,88,89]
Marginalized and underserved rural populations	Limited PPE, poor training, weak regulation, unsafe storage/disposal, low access to healthcare, poverty, limited political voice	Smallholder farms, plantations, informal agricultural work, contaminated water sources	Acute poisoning, chronic disease, economic burden, underreported illness	Some vulnerable groups may experience higher exposure while having limited access to protection	[5,26,64,90,91]
Non-farm residents near agricultural land	Proximity to sprayed fields, spray drift, volatilization, contaminated residential dust	Homes, schools, public spaces near farms	Respiratory symptoms, biomarker changes, possible chronic outcomes	Pesticide exposure may occur in both occupational and community settings	[25,44,74,93]

## Data Availability

No new data were created or analyzed in this study. Data sharing is not applicable to this article.
